# Contribution of *MUTYH* Variants to Male Breast Cancer Risk: Results From a Multicenter Study in Italy

**DOI:** 10.3389/fonc.2018.00583

**Published:** 2018-12-04

**Authors:** Piera Rizzolo, Valentina Silvestri, Agostino Bucalo, Veronica Zelli, Virginia Valentini, Irene Catucci, Ines Zanna, Giovanna Masala, Simonetta Bianchi, Alessandro Mauro Spinelli, Stefania Tommasi, Maria Grazia Tibiletti, Antonio Russo, Liliana Varesco, Anna Coppa, Daniele Calistri, Laura Cortesi, Alessandra Viel, Bernardo Bonanni, Jacopo Azzollini, Siranoush Manoukian, Marco Montagna, Paolo Radice, Domenico Palli, Paolo Peterlongo, Laura Ottini

**Affiliations:** ^1^Department of Molecular Medicine, Sapienza University of Rome, Rome, Italy; ^2^Genome Diagnostics Program, IFOM - The FIRC Institute of Molecular Oncology, Milan, Italy; ^3^Cancer Risk Factors and Lifestyle Epidemiology Unit, Institute for Cancer Research, Prevention and Clinical Network (ISPRO), Florence, Italy; ^4^Division of Pathological Anatomy, Department of Surgery and Translational Medicine, University of Florence, Florence, Italy; ^5^Institute for Maternal and Child Health IRCCS Burlo Garofolo, Trieste, Italy; ^6^Molecular Genetics Laboratory, Istituto Tumori Giovanni Paolo II, Bari, Italy; ^7^Dipartimento di Patologia, ASST Settelaghi and Centro di Ricerca per lo studio dei tumori eredo-familiari, Università dell'Insubria, Varese, Italy; ^8^Section of Medical Oncology, Department of Surgical and Oncological Sciences, University of Palermo, Palermo, Italy; ^9^IRCCS Ospedale Policlinico San Martino, Genoa, Italy; ^10^Department of Experimental Medicine, Sapienza University of Rome, Rome, Italy; ^11^Istituto Scientifico Romagnolo per lo Studio e la Cura dei Tumori (IRST), Meldola, Italy; ^12^Department of Oncology and Haematology, University of Modena and Reggio Emilia, Modena, Italy; ^13^Unità di Oncogenetica e Oncogenomica Funzionale, Centro di Riferimento Oncologico di Aviano (CRO), IRCCS, Aviano, Italy; ^14^Division of Cancer Prevention and Genetics, European Institute of Oncology (IEO), IRCCS, Milan, Italy; ^15^Unità di Genetica Medica, Dipartimento di Oncologia Medica ed Ematologia, Fondazione IRCCS Istituto Nazionale dei Tumori (INT), Milan, Italy; ^16^Immunology and Molecular Oncology Unit, Veneto Institute of Oncology IOV - IRCCS, Padua, Italy; ^17^Unità di Ricerca Medicina Predittiva: Basi molecolari Rischio genetico e Test genetici, Dipartimento di Ricerca, Fondazione IRCCS Istituto Nazionale Tumori (INT), Milan, Italy

**Keywords:** male breast cancer, genetic susceptibility, *BRCA1/2*, *MUTYH*, NGS, MUTYH-associated polyposis (MAP) syndrome, breast cancer risk

## Abstract

Inherited mutations in *BRCA1*, and, mainly, *BRCA2* genes are associated with increased risk of male breast cancer (MBC). Mutations in *PALB2* and *CHEK2* genes may also increase MBC risk. Overall, these genes are functionally linked to DNA repair pathways, highlighting the central role of genome maintenance in MBC genetic predisposition. *MUTYH* is a DNA repair gene whose biallelic germline variants cause MUTYH-associated polyposis (MAP) syndrome. Monoallelic *MUTYH* variants have been reported in families with both colorectal and breast cancer and there is some evidence on increased breast cancer risk in women with monoallelic variants. In this study, we aimed to investigate whether *MUTYH* germline variants may contribute to MBC susceptibility. To this aim, we screened the entire coding region of *MUTYH* in 503 *BRCA1/2* mutation negative MBC cases by multigene panel analysis. Moreover, we genotyped selected variants, including p.Tyr179Cys, p.Gly396Asp, p.Arg245His, p.Gly264Trpfs^*^7, and p.Gln338His, in a total of 560 MBC cases and 1,540 male controls. Biallelic *MUTYH* pathogenic variants (p.Tyr179Cys/p.Arg241Trp) were identified in one MBC patient with phenotypic manifestation of adenomatous polyposis. Monoallelic pathogenic variants were identified in 14 (2.5%) MBC patients, in particular, p.Tyr179Cys was detected in seven cases, p.Gly396Asp in five cases, p.Arg245His and p.Gly264Trpfs^*^7 in one case each. The majority of MBC cases with *MUTYH* pathogenic variants had family history of cancer including breast, colorectal, and gastric cancers. In the case-control study, an association between the variant p.Tyr179Cys and increased MBC risk emerged by multivariate analysis [odds ratio (OR) = 4.54; 95% confidence interval (CI): 1.17–17.58; *p* = 0.028]. Overall, our study suggests that *MUTYH* pathogenic variants may have a role in MBC and, in particular, the p.Tyr179Cys variant may be a low/moderate penetrance risk allele for MBC. Moreover, our results suggest that MBC may be part of the tumor spectrum associated with MAP syndrome, with implication in the clinical management of patients and their relatives. Large-scale collaborative studies are needed to validate these findings.

## Introduction

Male Breast Cancer (MBC) is a rare disease whose etiology appears to be associated with genetic factors. Inherited mutations in *BRCA1* and, mainly, *BRCA2*, predispose to MBC and account for up to 13% of all cases in the Italian population (1). Even though there is evidence supporting an association between increased MBC risk and pathogenic variants in *PALB2* and *CHEK2* (2–4), these two genes are unlikely to account for a substantial fraction of MBC cases. Thus, additional genes that may contribute to MBC genetic susceptibility need to be investigated.

*BRCA1, BRCA2, PALB2*, and *CHEK2* belong to or are functionally linked to the Homologous Recombination (HR) mechanism, one of the most important DNA Double-Strand Break (DSB) repair pathways, highlighting the central role of genome maintenance in MBC predisposition (5). Overall, the maintenance of genomic integrity is achieved by a coordinated interplay of different mechanisms of DNA repair, including Mismatch Repair (MMR), Nucleotide Excision Repair (NER) and Base Excision Repair (BER), in addition to DSB repair (6, 7). While dysregulation of DSB repair is known to play a relevant role in breast cancer (BC) pathogenesis, the involvement of other DNA repair pathways in BC is much less established.

*MUTYH* encodes a DNA glycosylase involved in BER, preventing 8-oxo-G:A mispairs generated by oxidative damage (8). Oxidative DNA damage, including 8-oxoG, may be due to hormonal metabolism and may contribute to BC susceptibility (9, 10). In this context, it is noteworthy that *BRCA1* and *BRCA2* are also involved in 8-oxoG repair (11), thus further supporting a possible role of BER and, more specifically, *MUTYH* in BC pathogenesis.

Biallelic (homozygous or compound heterozygous) *MUTYH* variants occur in 0.01–0.04% of European descent populations and cause MUTYH-associated polyposis syndrome (MAP), which predisposes patients to develop colorectal polyps and colorectal cancer (12–19). Monoallelic (heterozygous) *MUTYH* variants occur in 1–2% of European descent populations and are associated with an increased risk of colorectal cancer (14, 16–21). Several studies on extracolonic cancers in carriers of *MUTYH* variants have been performed (21–26). The association of *MUTYH* variants with malignancies other than colon cancer is less robust, especially when establishing cancer risks in heterozygous *MUTYH* individuals. Increased risks of bladder and ovarian cancers have been reported for biallelic mutation carriers, while slightly increased risks of gastric, hepatobiliary, endometrial, and breast cancer have been observed in monoallelic mutation carriers (27).

Overall, the association between *MUTYH* mutations and BC risk remains controversial, some studies have shown an increased BC risk among *MUTYH* mutation carriers, while others have not (22–26, 28–30). An increased risk of BC associated with biallelic and monoallelic variants of *MUTYH* has been reported in *BRCA1/2* mutation negative individuals (21–23, 26). A higher frequency of monoallelic *MUTYH* mutations in families with both breast and colorectal cancer has been also reported compared to general population (21). Recently, an increased BC risk has been also reported for women with the common p.Gln338His variant (31).

To date the possible association between *MUTYH* variants and MBC risk has not been investigated. MBC is recognized as being primarily a hormone-dependent malignancy and is widely accepted as an estrogen-driven disease specifically related to hyperestrogenism (32) thus, oxidative DNA damage, due to hormonal metabolism, may particularly contribute to BC susceptibility in men. In this context, impairment of MUTYH activity due to inactivating/pathogenic variants may contribute to increase MBC risk.

To assess if *MUTYH* germline variants may contribute to MBC susceptibility, we screened a large series of *BRCA1/2* mutation negative MBC patients by sequencing the entire *MUTYH* coding region. Furthermore, to explore whether *MUTYH* variants were significantly associated with MBC risk, we performed a case-control study of selected *MUTYH* variants.

## Patients and Methods

### Study Population

A total of 560 *BRCA1*/*2* mutation negative MBC cases and 1,540 male controls, enrolled in the frame of the ongoing Italian Multicenter Study on MBC (33), were included in the present study. For each MBC case, information on the main clinical-pathologic characteristics were collected as previously described (33, 34). Controls were male individuals without personal history of cancer, enrolled under research or clinical protocols, or blood donors. All controls were recruited in the same geographical area of cases. For each study participant, samples of blood or DNA from peripheral blood leukocytes were collected. DNA from blood samples was extracted and quantified as previously described (35). The study was approved by Local Ethical Committee (Sapienza University of Rome, Prot. 669/17) and informed consent for using information and biological samples was obtained from all participants to the study.

### *MUTYH* Gene Sequencing

A total of 503 MBC cases underwent next generation sequencing (NGS) of a custom panel of 50 cancer susceptibility genes including *MUTYH*. Briefly, paired-end libraries were prepared using the Nextera Rapid Capture Custom Enrichment kit (Illumina, San Diego, California, USA), pooled and loaded into a MiniSeq system (Illumina) for automated cluster generation, sequencing, and data analysis, including variant calling. Variant annotation and filtering was performed with Illumina Variant Studio Software version 2.2 against the human reference genome GRCh37. Variants were classified as pathogenic or likely pathogenic (collectively termed, pathogenic) according to the American College of Medical Genetics and Genomics (ACMG) recommendations (36). Briefly, variants were classified as pathogenic if they had a truncating, initiation codon or splice donor/acceptor effect or if pathogenicity was demonstrated by functional studies supportive of a damaging effect on the gene or gene product. All pathogenic variants were confirmed by double-stranded Sanger Sequencing (primer sequences are available upon request). Variants were named according to Human Genome Variation Society nomenclature (HGVS, http://www.hgvs.org).

### Genotyping Analysis

Genotyping analysis of five *MUTYH* variants, rs34612342 (c.536A>G; p.Tyr179Cys), rs36053993 (c.1187G>A; p.Gly396Asp), rs140342925 (c.734G>A; p.Arg245His), rs587780751 (c.933+3A>C; p.Gly264Trpfs^*^7), and rs3219489 (c.1014G>C; p.Gln338His), identified by NGS and selected because previously proposed to be associated with increased risk of extracolonic cancer, including BC, was performed by allelic discrimination real-time PCR, in an ABI 7500 fast real-time PCR instrument (Life Technologies, Carlsbad, California, USA), using commercially available TaqMan SNP genotyping assays (Life Technologies) and according to the manufacturer's instructions. The specific assay IDs used are: C_32911941_10 (rs36512342), C_27860250_10 (rs36053993), C_166223223_10 (rs140342925), C_362043726_10 (rs587780751), and C_27504565_10 (rs3219489). In each experiment, positive (cases for which genotype was confirmed by Sanger Sequencing) and negative (water) controls were always included. A total of 560 MBC cases, including the 503 cases analyzed by NGS, and 1,540 male controls were genotyped.

### Statistical Analysis

Chi-square test was performed in a case-case analysis in order to evaluate potential associations between pathogenic variants and specific clinical-pathologic characteristics.

The genotype frequency for each variant was evaluated in both series of cases and controls. The association between each variant and overall MBC risk was measured by the odds ratio (OR) and its corresponding 95% confidence interval (CI) by univariate logistic regression, and also by a multivariate analysis including adjustment for age, center and type of enrolment. A *p*-value < 0.05 was considered statistically significant. All the analyses were performed using STATA version 13.1 statistical program.

## Results

### Clinical-Pathologic Characteristics of MBC Cases

The study population consisted of 560 *BRCA1*/*2* mutation negative MBC cases, enrolled in the frame of the ongoing Italian Multicenter Study on MBC. Overall, mean age at first BC diagnosis was 61.8 years (range 22–91 years); 91 cases (16.2%) reported first-degree family history of breast and/or ovarian cancer (BC/OC), 247 cases (44.1%) had first-degree family history of cancer and 101 cases (18%) had a personal history of cancer in addition to BC, mostly colorectal and prostate cancer. The majority of male breast tumors were invasive ductal carcinomas (85.9%), estrogen receptor positive (ER+, 94.2%), progesterone receptor positive (PR+ 88.4%), and HER2 negative (79.2%).

### *MUTYH* Gene Sequencing in MBC Cases

The entire coding region of *MUTYH* was screened in 503 *BRCA1/2* mutation negative MBC cases, by a custom multigene panel using NGS technologies. *MUTYH* variants detected are shown in Table 1. p.Tyr179Cys and p.Gly396Asp variants were the most frequently detected pathogenic variants and were identified in 1.6 and 1.0% of the MBC cases, respectively. The common variant p.Gln338His was identified in 41.7% of the MBC cases (Table 1).

**Table 1:** *MUTYH* variants detected by NGS in 503 *BRCA1/2* mutation negative MBC cases ^a^.

**^b^Location**	**Nucleotide change**	**Protein change**	**Variant type**	**dbSNP ID**	**Frequency *N* (%)**
Exon 2	c.37 G>A	p.Ala13Thr	Missense	rs375349172	1 (0.2%)
Exon 2	c.64G>A	p.Val22Met	Missense	rs3219484	40 (8.0%)
**Exon 7**	**c.536A**>**G**	**p.Tyr179Cys**	**Missense**	**rs34612342**	**8 (1.6%)**
Exon 9	c.694A>T	p.Thr232Ser	Missense	rs587782351	1 (0.2%)
**Exon 9**	**c.721C**>**T**	**p.Arg241Trp**	**Missense**	**rs34126013**	**1 (0.2%)**
**Exon 9**	**c.734G**>**A**	**p.Arg245His**	**Missense**	**rs140342925**	**1 (0.2%)**
Exon 10	c.919C>T	p.Arg307Trp	Missense	rs759822330	1 (0.2%)
**IVS 10**	**c.933**+**3A**>**C**^c^	p.Gly264Trpfs*7	**Frameshift**	**rs587780751**	**1 (0.2%)**
Exon 12	c.1014G>C	p.Gln338His	Missense	rs3219489	210 (41.7%)
Exon 12	c.1037C>G	p.Ser346Trp	Missense	rs587778538	1 (0.2%)
**Exon 13**	**c.1187G**>**A**	**p.Gly396Asp**	**Missense**	**rs36053993**	**5 (1.0%)**
Exon 13	c.1258C>A	p.Leu420Met	Missense	rs144079536	4 (0.8%)
Exon 13	c.1276C>T	p.Arg426Cys	Missense	rs150792276	2 (0.4%)
Exon 16	c.1544 C>T	p.Ser515Phe	Missense	rs140118273	6 (1.2%)

a *NGS, Next Generation sequencing; MBC, Male Breast Cancer*.

b *Pathogenic variants are shown in bold text*.

c *This variant affects a splicing site and causes the skipping of exon 10 that leads to a premature stop codon*.

Overall, pathogenic variants were identified in 15 (3.0%) MBC cases (Table 2), 14 cases were carriers of monoallelic (heterozygous) pathogenic variants and one case was carrier of the biallelic p.Tyr179Cys/p.Arg241Trp (compound heterozygous) pathogenic variants. The majority of MBC cases with *MUTYH* pathogenic variants had family history of cancer including breast, colorectal, and gastric cancers (Table 2). In particular, the biallelic *MUTYH* pathogenic variant carrier was a man diagnosed with BC at 51 years of age who developed colon cancer, with phenotypic manifestation of adenomatous polyposis, at early age (41 years) and had a first-degree relative affected by melanoma at young age (26 years). With the exception of this case, clinical features of the other MBC patients with *MUTYH* pathogenic variants did not suggest a MAP phenotype.

**Table 2:** Personal and family history of cancer in MBC cases with germline *MUTYH* pathogenic variants^a^.

**ID**	**Variant^b^**	**Personal history of cancer (age)**	**First-degree family history of cancer (age)**
#40	c.1187G>A (p.Gly396Asp)	Breast (55)	Prostate (85)
#61	c.1187G>A (p.Gly396Asp)	Breast (70)	Colorectal (56)
#138	c.1187G>A (p.Gly396Asp)	Breast (66)	Breast (50)
#153	c.1187G>A (p.Gly396Asp)	Breast (51)	–
#321	c.1187G>A (p.Gly396Asp)	Breast (75)	–
#146	c.536A>G (p.Tyr179Cys)	Breast (80)	Breast (45); Gastric (54)
#236	c.536A>G (p.Tyr179Cys)	Breast (45)	Breast (58); Colorectal (58)
#317	c.536A>G (p.Tyr179Cys)	Breast (67); Prostate (68)	–
#341	c.536A>G (p.Tyr179Cys)	Breast (72)	Breast (70); Esophageus (76)
#352	c.536A>G (p.Tyr179Cys)	Breast (63)	–
#376	c.536A>G (p.Tyr179Cys)	Breast (61)	Gastric (43); Liver (67)
#478	c.536A>G (p.Tyr179Cys)	Breast (82)	Colon (50)
#257	c.933+3A>C p.Gly264Trpfs*7)	Breast (72)	3 Breast (72,76, na)^a^
#358	c.734G>A (p.Arg245His)	Breast (79)	Breast (65); Gastric (69)
#227	c.536A>G (p.Tyr179Cys); c.721C>T (p.Arg241Trp)	Breast (51); Colon (41)	Melanoma (26)

a *MBC, Male Breast Cancer; na, not available*.

b *Variants nomenclature in according to RefSeq NM_001128425.1, NP_001121897.1*.

Overall, comparison of the clinical-pathologic characteristics between *MUTYH* pathogenic variant carriers and non-carriers did not show any statistically significant differences (Table 3).

**Table 3:** Clinical-pathologic characteristics of *MUTYH* pathogenic variant carriers and non-carriers.

**Characteristics^a^**	***MUTYH*** **variant carriers (*****N*** = **15)**		**Non-carriers (*****N*** = **488)**		***p*-value**
	***N***	**%**	***N***	**%**
**Mean age at diagnosis** ± **SD (range)**	65.9 ± 11.4 (45–82)		61.7 ± 12 (22–91)		0.2
**FIRST-DEGREE FAMILY HISTORY OF BC/OC**^b^					
Negative	13	86.6	411	84.4
Positive	2	13.4	76	15.6	1
**FIRST-DEGREE FAMILY HISTORY OF CANCER**					
Negative	6	40.0	274	56.3
Positive	9	60.0	213	43.7	0.3
**PERSONAL HISTORY OF CANCER IN ADDITION TO BC**					
Negative	12	80.0	396	81.1
Positive	3	20.0	92	8.9	1
**TUMOR HISTOTYPE**					
Invasive ductal carcinoma	13	92.9	342	83
*In situ* ductal carcinoma	1	7.1	36	8.7
Other	–	–	34	8.3	0.9
**TNM STAGE**					
0	1	7.1	33	9.8
1	6	42.9	152	45.4
2	6	42.9	95	28.4
3–4	1	7.1	55	16.4	0.7
**HISTOLOGIC GRADE**					
1	1	7.7	44	13.3
2	9	69.2	198	59.8
3	3	23.1	89	26.9	0.9
**LYMPH NODE STATUS**					
Negative	8	61.5	213	63.2
Positive	5	38.5	124	36.8	1
**ER**^b^ **STATUS**					
Negative	1	8.3	23	6.1
Positive	11	91.7	353	93.9	0.5
**PR**^b^ **STATUS**					
Negative	1	9.1	43	11.5
Positive	10	90.9	331	88.5	1
**HER2**^b^ **STATUS**					
Negative	11	91.7	236	80.0
Positive	1	8.3	59	20.0	0.5
**Ki67/MIB1 STATUS**					
Low	2	28.6	172	58.9
High	5	71.4	120	41.1	0.1

a *Some data for each pathologic characteristic are not available*.

b *BC, breast cancer; OC, ovarian cancer; ER, Estrogen receptor; PR, Progesterone receptor; HER2, human epidermal growth factor receptor 2*.

### Genotyping Analysis of Selected *MUTYH* Variants in MBC Cases and Controls

*MUTYH* pathogenic variants, including p.Tyr179Cys (rs34612342), p.Gly396Asp (rs36053993), p.Arg245His (rs140342925), p.Gly264Trpfs^*^7 (rs587780751), and the common variant p.Gln338His (rs3219489), were genotyped in 560 cases and 1,540 male controls. Overall, pathogenic variants were detected at significantly higher frequency (*p* = 0.04) in MBC cases (15/560 2.7%) than in controls (21/1540, 1.3%).

The distribution of genotype frequencies and the estimates for the association between each genotyped variant and overall MBC risk are summarized in Table 4. Significant differences in the distribution of genotypes between MBC cases and controls emerged for p.Tyr179Cys (rs34612342) variant. The analysis of the genotype-specific risks showed that men with heterozygous genotype for *MUTYH* p.Tyr179Cys variant were at increased BC risk both in the univariate (OR = 5.56; 95%CI:1.67–18.55; *p* = 0.005) and in the multivariate analysis (OR = 4.54; 95%CI:1.17–17.58; *p* = 0.028). No statistically significant differences in genotype distribution between case and controls emerged for the other variants analyzed.

**Table 4:** Distribution of 560 *BRCA1/2* negative MBC cases and 1,540 controls according to genotype frequencies and MBC risk estimates for selected *MUTYH* variants^a^.

				**Univariate analysis**		**Multivariate analysis****^b^**	
**Variant**	**Genotype**	**Cases *N* (%)**	**Controls *N* (%)**	**OR (95% CI)**	***p*-value^c^**	**OR (95% CI)**	***p*-value^c^**
**p.Tyr179Cys** (rs34612342)	AA	552 (98.57)	1536 (99.74)			
	AG	8 (1.43)	4 (0.26)	**5.56 (1.67–18.55)**	**0.005**	**4.54 (1.17–17.58)**	**0.028**
**p.Arg245His** (rs140342925)	GG	559 (99.8)	1540 (100)			
	GA	1 (0.2)	–	–	–	–	–
p.Gly264Trpfs*7 (rs587780751)	AA	559 (99.8)	1539 (99.94)			
	AC	1 (0.2)	1 (0.06)	2.75 (0.17–44)	0.455	0.94(0.04–19.95)	0.97
**p.Gln338His** (rs3219489)	GG	327 (58.4)	931(60)			
	GC	203 (36.2)	526 (34.2)	1 (0.89–1.34)	0.36	1.2 (0.95–1.5)	0.12
	CC	30 (5.4)	83 (5.4)	2.1 (0.66–1.59)	0.89	1.2 (0.76–2)	0.37
**p.Gly396Asp** (rs36053993)	GG	555 (99.1)	1524 (99)			
	GA	5 (0.9)	16 (1)	0.86 (0.31–2.35)	0.77	0.58 (0.16–2.14)	0.42

a *MBC, Male breast Cancer; OR, Odds Ratio; 95% CI, 95% confidence interval*.

b *ORs and 95% CI for specific genotypes were calculated using logistic regression models adjusted for age, center and type of enrolment*.

c *p-values < 0.05 in bold text*.

## Discussion

In this study, we aimed to evaluate the contribution of *MUTYH* variants in MBC susceptibility. To this purpose, we obtained NGS data of the entire coding region of *MUTYH* from a large series of *BRCA1/2* mutation negative MBC cases, from the ongoing Italian Multicenter Study on MBC, and further genotyped selected variants in a case-control study. To date, there is contrasting evidence on the impact of *MUTYH* pathogenic variants on risk of BC in women and, to the best of our knowledge, no study has been performed in MBC.

By NGS, we identified 15 MBC patients (3.0%) with germline *MUTYH* pathogenic variants, including one biallelic and 14 monoallelic variant carriers. The MBC patient with biallelic *MUTYH* pathogenic variants was affected by colorectal cancer at early age with phenotypic manifestation of adenomatous polyposis. Thus, our results allowed a molecular diagnosis of MAP. To the best of our knowledge, to date, only another MBC case has been reported with MAP syndrome (23). Taking into account the rarity of both MBC and MAP, the occurrence of MBC in MAP patients may underline a possible common genetic pathway and suggest that MBC could be considered a MAP-related malignancy.

Overall, *MUTYH* monoallelic pathogenic variants, including p.Tyr179Cys, p.Gly396Asp, p.Arg245His, and p.Gly264Trpfs^*^7, were found with a frequency of 2.8% in our MBC series. p.Tyr179Cys and p.Gly396Asp were the most frequently variants detected and were identified in 2.4% of the cases. Published data showed that these two variants are the most frequent pathogenic variants in populations of European origin and account for 50 to 90% of *MUTYH* pathogenic variants identified in MAP patients (13, 14, 37, 38). The p.Arg245His variant was identified in a MBC patient with family history of breast and gastric cancers. This variant has been reported strongly associated with familial colorectal cancer (23, 39), and has also been identified in patients with suspected Lynch Syndrome and in a patient with gastric cancer (23, 40). The p.Gly264Trpfs^*^7 variant was identified in a MBC patient, from North-East of Italy, where it occurs as a founder mutation accounting for about 15.0% of the *MUTYH* pathogenic variants identified in MAP patients (41). By contrast, this variant has been reported with lower frequency, ranging from 1.0 to 8.0%, in MAP patients from other populations of Caucasian ethnicity (23, 41–47).

To investigate whether MBC arising in *MUTYH* pathogenic variant carriers may be characterized by specific features, we compared clinical-pathologic characteristics between carriers and non-carriers. No statistically significant association emerged for any of the clinical features tested. However, the great majority of MBC patients with *MUTYH* pathogenic variants had family history of cancer, including, breast, colorectal, and gastric cancers. These findings, if confirmed by additional data, may be useful in decisions concerning clinical management of patients and their families.

To further investigate the role of *MUTYH* in MBC, we evaluated the risk of MBC associated with selected *MUTYH* variants previously proposed to be associated with increased cancer risk, including BC risk (21, 27, 31), by performing a case-control study. Among the pathogenic variants examined, the p.Tyr179Cys variant was associated with an increased MBC risk (OR = 4.54, 95%CI = 1.17–17.58). A higher frequency of p.Tyr179Cys has been reported in families with both breast and colorectal cancer compared to the general population (21), but an association between p.Tyr179Cys variant and increased BC risk has not been observed (25, 26, 28, 30). Our results, suggest that p.Tyr179Cys variant may be a low/moderate penetrance risk allele for BC in men. This variant, located at 8-oxo-G binding site, causes major structural protein changes and a reduction in functionality (48, 49). Thus, oxidative DNA damage due to hormonal metabolism, like estrogen-induced 8-oxo-dG generation, may particularly contribute to MBC susceptibility, as BC in men is primarily a hormone-dependent tumor, specifically related to hyperestrogenism. Furthermore, it can be hypothesized that MBC, unencumbered by the many confounding factors that exist in female BC (i.e., reproductive factors and high frequency) might facilitate the identification of genetic factors and molecular mechanisms that may influence BC risk in general (50).

We also assessed whether the common p.Gln338His variant, reported to increase BC risk in women (31), was associated with MBC risk. We did not observe any significant differences in p.Gln338His genotypes distribution between MBC cases and controls inconsistent with a possible role of this variant in MBC risk. The other common variant, p.Val22Met, has not been reported to be associated with cancer risk (51–53) and was not examined in this study.

Overall, we observed that the majority of MBC patients with pathogenic *MUTYH* variants have first-degree family history of cancers. This raises the question of whether *MUTYH* variants, especially the Tyr179Cys variant, may be associated with MBC risk only, or with the risk of familial or multi-syndromic diseases, including MBC. Further clinical/phenotype assessments and detailed statistical analyses would be useful in future studies to answer this question.

In conclusion, our study suggests that *MUTYH* pathogenic variants may have a role in MBC, in particular, p.Tyr179Cys variant may be a low/moderate penetrance risk allele for MBC. Our findings also suggest that MBC may be part of the tumor spectrum associated with MAP syndrome, with implications in the clinical management of the patients and their relatives.

Although we have a large series of MBC cases, this study may be underpowered to detect smaller risk effects and large-scale collaborative studies are needed to investigate any possible association with rarer variants and to have a more comprehensive examination and characterization of the link between *MUTYH* variants and MBC risk.

## Data Availability Statement

Datasets are available on request. The raw data supporting the conclusions of this manuscript will be made available by the authors, without undue reservation, to any qualified researcher.

## Authors Contributions

PiR drafted the manuscript, performed NGS and statistical analyses and interpreted the results. VS performed genotyping and statistical analyses, and interpreted the results. AB and IC performed genotyping analysis. VZ and VV performed NGS analysis. IZ, GM, SB AS, ST, MT, AR, LV, AC, DC, LC, AV, BB, JA, SM, MM, PaR, and DP recruited samples and collected clinicalpathologic data. PP contributed to study design, recruited samples and collected clinical pathologic data. LO conceived, designed and coordinated the study, and drafted the manuscript. All authors reviewed, edited, and approved the manuscript for publication.

### Conflict of Interest Statement

The authors declare that the research was conducted in the absence of any commercial or financial relationships that could be construed as a potential conflict of interest.

## Abbreviations

ACMG: American College of Medical Genetics and Genomics
BC: breast cancer
BER: base excision repair
DSB: double-strand break
ER: estrogen receptor
HER2: human epidermal growth factor receptor 2
HGVS: Human Genome Variation Society
HR: homologous recombination
MAP: MUTYH-associated polyposis
MBC: male breast cancer
MMR: mismatch repair
NER: nucleotide excision repair
NGS: next generation sequencing
OC: ovarian cancer
OR: Odds Ratio
PR: progesterone receptor
CI: confidence interval.
